# Colchicine Use Is Associated with Shorter ICU Stay and Lower FFP Transfusion Requirements After CABG

**DOI:** 10.3390/jcdd13100527

**Published:** 2026-10-20

**Authors:** Mehmet Burak Gulcan, Mehmet Ali Kaygın, Aycan Mutlu Yaganoglu, Ziya Yıldız, Abdulselam Karaduman, Suleyman Aycan, Ozgur Dag

**Affiliations:** 1 Department of Cardiovascular Surgery, Ankara Yıldırım Beyazıt University Yenimahalle Training and Research Hospital, 06370 Ankara, Türkiye; 2 Department of Cardiovascular Surgery, Erzurum City Hospital, 25240 Erzurum, Türkiye; 3 Department of Zootechnics, Faculty of Agriculture, Ataturk University, 25240 Erzurum, Türkiye; 4 Department of Congenital Cardiovascular Surgery, Mehmet Akif Ersoy Chest and Cardiovascular Surgery Research and Training Hospital, 34303 Istanbul, Türkiye

**Keywords:** coronary artery bypass, anti-inflammatory agents, inflammation, colchicine

## Abstract

Background: Colchicine has demonstrated anti-inflammatory and cardiovascular benefits in patients with coronary heart disease. However, evidence regarding its effects on postoperative inflammation and early clinical outcomes following coronary artery bypass grafting (CABG) remains limited. Objective: We aimed to evaluate the association between perioperative low-dose colchicine use and postoperative clinical outcomes, transfusion requirements, complications, and inflammatory markers in patients undergoing elective on-pump CABG. Methods: This retrospective study included 151 patients who underwent elective on-pump CABG: 81 received colchicine (0.5 mg once daily for 3 days preoperatively and 10 days postoperatively) and 70 did not receive colchicine in the past period. The primary clinical outcomes included ICU and total hospital length of stay, erythrocyte and fresh frozen plasma (FFP) transfusion requirements, wound complication, and surgical revision. Postoperative day 5 laboratory parameters included C-reactive protein, white blood cell (WBC) count, systemic immune-inflammation index (SII), and albumin. Between-group comparisons were performed using Student’s or Welch’s *t* tests and Pearson’s chi-square or Fisher’s exact tests, as appropriate. Results: Baseline age and sex distribution were similar between the groups. The colchicine group had a significantly shorter ICU stay than the control group (4.28 ± 1.95 vs. 5.83 ± 3.04 days; mean difference, −1.54 days; 95% CI, −2.38 to −0.71; *p* < 0.001). FFP transfusion requirements were also significantly lower in the colchicine group (3.49 ± 1.24 vs. 8.13 ± 3.97 units; mean difference, −4.63 units; 95% CI, −5.62 to −3.65; *p* < 0.001). Postoperative WBC counts were lower with colchicine (9.44 ± 3.31 vs. 10.78 ± 4.04 × 10^9^/L; *p* = 0.027). Total hospital stay, erythrocyte transfusion requirements, other inflammatory parameters, wound complication rates, and surgical revision rates did not differ significantly between the groups. Conclusions: Perioperative low-dose colchicine was found to be in relation with shorter ICU stays, lower FFP transfusion requirements, and reduced postoperative WBC counts following elective on-pump CABG according to this retrospective study. Prospective randomized studies are required to confirm these findings and to determine whether colchicine improves early postoperative outcomes.

## 1. Introduction

Cardiovascular disease (CVD) is the leading cause of mortality worldwide, and it is estimated to account for more than 22 million deaths by 2030 [1]. Coronary heart disease (CHD) is a major contributor to CVD-related mortality and morbidity, followed by stroke [1]. Ischemic heart disease (IHD) alone is one of the leading causes of death worldwide, second only to neonatal disorders [2]. Myocardial infarction (MI) is the most important clinical manifestation of CHD and one of the leading causes of death and disability worldwide [3].

Revascularization has become a major treatment modality for both acute and chronic CHD [4,5]. Percutaneous coronary intervention (PCI) and coronary artery bypass grafting (CABG) are both widely accepted revascularization strategies for patients with CHD [4,5,6]. Although revascularization is important and often essential, it should be complemented by pharmacological treatments, including antiplatelet and lipid-lowering therapies [4,5]. Furthermore, anti-inflammatory agents such as colchicine are currently included in guideline recommendations, particularly for patients with atherosclerotic CHD [4,5].

In 2019, the Colchicine Cardiovascular Outcomes Trial (COLCOT) demonstrated that low-dose colchicine was associated with a lower risk of cardiovascular events in patients with a history of MI [7]. In 2020, the Low-Dose Colchicine 2 (LoDoCo2) trial showed that low-dose colchicine was associated with improved cardiovascular outcomes in patients with chronic CHD [8]. Subsequently, colchicine was incorporated into the perioperative medical management of selected patients undergoing CABG. Moreover, studies have demonstrated the anti-inflammatory effects of colchicine in patients undergoing CABG, primarily as reflected by improvements in biochemical parameters [9,10,11].

However, few studies have investigated the effects of colchicine on clinical outcomes and postoperative morbidity. Our study aimed to address this gap in the literature. In this retrospective study, we evaluated total length of hospital stay, length of intensive care unit stay, the number of erythrocyte and fresh frozen plasma transfusions, age, sex, wound complication, surgical revision, and routinely measured inflammatory parameters on postoperative day 5, including C-reactive protein (CRP), white blood cell (WBC) count, albumin, platelet-to-lymphocyte ratio (PLR), and neutrophil-to-lymphocyte ratio (NLR). These parameters were analyzed and compared between patients who received colchicine and those who did not. Our aim is to contribute to the literature with colchicine-related clinical outcomes which have not been investigated before.

## 2. Materials and Methods

This retrospective clinical study was approved by the Scientific Research Ethics Committee of the University of Health Sciences, Erzurum City Hospital (decision no. 2025/05-134) and was conducted in accordance with the ethical principles outlined in the Declaration of Helsinki. The study included patients who underwent elective on-pump coronary artery bypass grafting (CABG) performed by the same surgical, anesthesia, perfusion, and intensive care unit (ICU) teams.

Seventy patients who underwent CABG during the one-year period from October 2021 to October 2022 were sequentially selected to form the control group. These patients did not receive perioperative colchicine treatment. Beginning in September 2023, based on contemporary guidelines and the available literature, colchicine was incorporated into the routine perioperative medical treatment of patients undergoing CABG in the Department of Cardiovascular Surgery at Erzurum City Hospital. Colchicine was administered orally at a dose of 0.5 mg once daily for three days before surgery and for ten days after surgery, in addition to standard antiplatelet and lipid-lowering therapy. Eighty-one patients who underwent CABG during the one-year period from January 2024 to January 2025 were sequentially selected to form the colchicine group.

Demographic, clinical, and laboratory data were retrospectively obtained from archived medical records and the hospital’s electronic information system. The collected variables included age, sex, total length of hospital stay, length of ICU stay, number of erythrocyte suspension and fresh frozen plasma units transfused, surgical wound complication, and the need for surgical revision. Routinely measured inflammatory and laboratory parameters obtained on postoperative day 5 were also recorded, including C-reactive protein (CRP), white blood cell count, serum albumin level, platelet-to-lymphocyte ratio, and neutrophil-to-lymphocyte ratio.

Patients who underwent off-pump CABG or emergency surgery were excluded. Additional exclusion criteria were in-hospital mortality, renal failure, hepatic failure, the use of an intra-aortic balloon pump (IABP) or extracorporeal membrane oxygenation (ECMO), and the need for an additional surgical procedure because of cannulation-related complications. Patients requiring prolonged postoperative mechanical ventilation were also excluded. After the eligibility criteria had been applied, patients were selected from the hospital’s archived medical records and electronic database.

All CABG procedures were performed through a median sternotomy under general anesthesia by the same surgical and anesthesia teams. Cardiopulmonary bypass was established in all patients using standard aortic and right atrial cannulation. Myocardial protection was achieved using antegrade administration of del Nido cardioplegia directly into the aortic root. All procedures were performed with the same perfusion team. Complete surgical revascularization was performed according to the distribution of clinically significant coronary artery stenoses identified on preoperative coronary angiography. Following surgery, all patients were transferred to the cardiovascular surgery ICU and were extubated on the day of surgery. There was no big difference in patient follow-up protocols between the years included data collection. The conventional CABG procedure, ICU follow-up and protocols were same in these two separate periods. Acetylsalicylic acid (100 mg/day) was continued up to the time of surgery in all patients.

Transfusion practice was in line with the 2017 EACTS/EACTA patient blood management guidelines [12]. Fresh frozen plasma (FFP) was not administered prophylactically. It was given for persistent non-surgical bleeding, bleeding related to coagulation factor deficiency, or heparin resistance before cardiopulmonary bypass. Packed red blood cells (PRBCs) were transfused according to the patient’s clinical condition, including hemodynamic stability, tachyarrhythmia, and ECG changes; in the postoperative ICU period, transfusion was generally considered when the hemoglobin level fell below 8 g/dL. The study was conducted in Erzurum, Turkey, at an altitude of 1853 m, which may result in a greater need for PRBC replacement owing to the higher altitude. Additionally, 2017 EACTS guideline [12] recommendations were accounted for in both of the groups. The latter guideline was published in October 2024, and protocol changes regarding transfusions did not totally represent a significant difference between the two groups [13].

## 3. Statistical Analysis

Statistical analyses were performed using IBM SPSS Statistics for Windows, version 26.0 (IBM Corp., Armonk, NY, USA). The analysis included 151 patients, comprising 81 patients who received colchicine (colchicine group) and 70 who did not (control group). All statistical tests were two-sided, and a *p* value < 0.05 was considered statistically significant. Because the analyses were exploratory and no multiplicity adjustment was planned, *p* values were not adjusted for multiple comparisons.

Continuous variables were summarized as mean ± standard deviation (SD), whereas categorical variables were presented as numbers and percentages. Between-group comparisons of continuous variables were performed using the independent-samples Student’s *t* test. Homogeneity of variances was assessed using Levene’s test. When Levene’s test indicated unequal variances (*p* < 0.05), Welch’s *t* test with Satterthwaite-adjusted degrees of freedom was reported; otherwise, the equal-variance Student’s *t* test was used. Between-group mean differences are reported as colchicine minus control values, together with 95% confidence intervals (CIs).

Categorical variables were compared using Pearson’s chi-square test when expected cell frequencies were adequate. Fisher’s exact test was used when the assumptions of the chi-square test were not met. Exploratory sex-stratified comparisons within each treatment group were performed using independent-samples *t* tests with the same variance-selection procedure. Associations among continuous variables were evaluated separately in the colchicine and control groups using Pearson’s product–moment correlation coefficients with pairwise deletion of missing observations. Correlation coefficients (r) and two-sided *p* values are reported.

## 4. Results

A total of 151 patients were included: 81 in the colchicine group and 70 in the control group. Age was available for 80 patients in the colchicine group and for all 70 controls. The groups were similar with respect to age (62.73 ± 8.94 vs. 63.60 ± 10.34 years; mean difference, −0.88 years; 95% CI, −3.99 to 2.24; *p* = 0.579) and sex distribution (male: 85.0% vs. 74.3%; *p* = 0.102).

The mean intensive care unit stay was significantly shorter in the colchicine group than in the control group (4.28 ± 1.95 vs. 5.83 ± 3.04 days). Because variances were unequal, Welch’s *t* test was used (t = −3.65, df = 114.34; mean difference, −1.54 days; 95% CI, −2.38 to −0.71; *p* < 0.001). Total hospital stay was numerically longer in the colchicine group, but the difference did not reach statistical significance (27.10 ± 8.66 vs. 24.53 ± 8.13 days; mean difference, 2.57 days; 95% CI, −0.14 to 5.28; *p* = 0.063) (Table 1).

Fresh frozen plasma requirements were markedly lower in the colchicine group (3.49 ± 1.24 vs. 8.13 ± 3.97 units). Welch’s *t* test showed a mean between-group difference of −4.63 units (95% CI, −5.62 to −3.65; t = −9.39, df = 80.58; *p* < 0.001). In contrast, erythrocyte transfusion requirements did not differ significantly between groups (5.49 ± 2.70 vs. 5.89 ± 2.85 units; mean difference, −0.39 units; 95% CI, −1.28 to 0.50; *p* = 0.387) (Table 1).

On postoperative day 5, the colchicine group had a significantly lower white blood cell count than the control group (9.44 ± 3.31 vs. 10.78 ± 4.04 × 10^9^/L; mean difference, −1.34 × 10^9^/L; 95% CI, −2.52 to −0.16; *p* = 0.027). No significant between-group differences were observed in C-reactive protein, neutrophil-to-lymphocyte ratio, platelet-to-lymphocyte ratio, or albumin (all *p* > 0.05) (Table 1).

Wound complications occurred in 19 patients (23.5%) in the colchicine group and 26 (37.1%) in the control group. Although the absolute rate was lower with colchicine, the difference did not reach statistical significance (χ^2^ = 3.36, *p* = 0.067). Surgical revision rates were comparable (6.2% vs. 5.7%; Fisher’s exact *p* = 1.000) (Table 2).

Within-group exploratory comparisons showed no statistically significant sex-based differences in any continuous outcome or postoperative laboratory parameter in either the colchicine or the control group when the variance-selection rule specified above was applied (all *p* > 0.05). These findings should be interpreted cautiously because the female subgroups were small (n = 12 in the colchicine group and n = 18 in the control group) (Table 3).

In the colchicine group, intensive care unit stay correlated positively with total hospital stay (r = 0.472, *p* < 0.001), erythrocyte transfusion (r = 0.500, *p* < 0.001), and fresh frozen plasma transfusion (r = 0.335, *p* = 0.002), and inversely with albumin (r = −0.282, *p* = 0.011). Total hospital stay correlated positively with erythrocyte transfusion (r = 0.470, *p* < 0.001), fresh frozen plasma transfusion (r = 0.304, *p* = 0.006), and CRP (r = 0.399, *p* < 0.001), and inversely with albumin (r = −0.389, *p* < 0.001). Erythrocyte and fresh frozen plasma transfusion requirements were positively correlated (r = 0.529, *p* < 0.001). Erythrocyte transfusion was inversely correlated with albumin (r = −0.455, *p* < 0.001) (Table 4).

In the control group, intensive care unit stay correlated positively with total hospital stay (r = 0.550, *p* < 0.001), erythrocyte transfusion (r = 0.252, *p* = 0.035), fresh frozen plasma transfusion (r = 0.260, *p* = 0.030), WBC (r = 0.429, *p* < 0.001), and NLR (r = 0.401, *p* < 0.001), and inversely with albumin (r = −0.253, *p* = 0.035). Erythrocyte and fresh frozen plasma transfusion requirements were positively correlated (r = 0.486, *p* < 0.001); both were inversely correlated with albumin (r = −0.419, *p* < 0.001 and r = −0.457, *p* < 0.001, respectively) (Table 5).

## 5. Discussion

The autumn crocus plant was used to treat swelling and pain in ancient Egypt and ancient Greece [14]. Galen of Pergamon mentioned the use of colchicine for the treatment of gout [14]. Alexander of Tralles was the first to describe the gastrointestinal side effects of colchicine during the Byzantine period [14]. Colchicine, which is derived from the autumn crocus plant, has traditionally been used to treat gout and familial Mediterranean fever (FMF) [15]. Furthermore, colchicine is used to treat inflammatory and fibrotic conditions such as Behçet’s disease (BD) and pericarditis [15]. The primary mechanism of action of colchicine involves the inhibition of microtubule polymerization [15]. Microtubules play important roles in chemokine and cytokine secretion, intracellular trafficking, and cell migration [15]. Colchicine exerts its anti-inflammatory effects through several mechanisms, including the inhibition of inflammasome activation and interleukin-1 beta (IL-1β) production, as well as the suppression of neutrophil function [15]. Colchicine has gradually become an integral component of pericarditis treatment, and its addition to conventional therapy provides clinical benefits, including lower recurrence rates [16]. Accordingly, current guidelines include colchicine in the conventional treatment of both acute and recurrent pericarditis [17,18].

In 2013, Nidorf et al. [19] demonstrated, in the Low-Dose Colchicine (LoDoCo) trial, that low-dose colchicine reduced the risk of cardiovascular events in patients with stable CHD. They proposed that colchicine targets inflammation within unstable atherosclerotic plaques [19]. In 2019, Tardif et al. [7] showed, in the COLCOT, that colchicine was associated with fewer cardiovascular events in patients with a history of MI, without altering biochemical inflammatory markers. Subsequently, Nidorf et al. [8] reported the results of the LoDoCo2 trial. Although routine inflammatory markers were not assessed, their study confirmed that low-dose colchicine was associated with fewer cardiovascular events in patients with chronic CHD [8]. In contrast, Tong et al. [20] reported opposing results in patients with acute CHD who received colchicine during a 12-month follow-up period. Their findings indicated that colchicine provided no clinical benefit over 12 months and that patients receiving colchicine had higher mortality rates [20]. Colchicine is still not recommended as a routine medication for secondary prevention after CABG [21]. However, the latest American Heart Association (AHA) guideline, consistent with European guidelines, recommends low-dose colchicine for patients with acute CHD with class 2B [4,5,22].

Particularly since its inclusion in guidelines for the treatment of CHD, the literature on the relationship between colchicine and CABG has begun to expand. In contrast to Tardif et al. [7], Pan et al. [11] demonstrated that colchicine was associated with lower levels of inflammatory markers. However, Tardif et al. [7] investigated patients with a history of MI, whereas Pan et al. [11] studied patients who underwent non-CABG cardiac surgery. Furthermore, Gulcan et al. [23] recently reported favorable morbidity outcomes associated with perioperative colchicine use in patients undergoing valvular heart surgery. In this context, colchicine may attenuate surgery-related inflammation. Similarly, in our study of patients who underwent CABG, WBC counts were significantly lower in the colchicine group. Regarding inflammation, our findings appear to be consistent with those reported by Pan et al. [11]. They also demonstrated that colchicine was associated with improved cardioprotection, as indicated by lower postoperative CK-MB and troponin levels [11]. Furthermore, their study included a broader range of inflammatory markers, such as IL-6 and procalcitonin [11]. The assessment of these markers was not feasible in our study because of its retrospective design. Therefore, we focused on routinely measured inflammatory markers, including CRP, WBC, NLR, PLR, and albumin. Colchicine was associated with lower WBC counts. However, none of the other inflammatory markers differed significantly between the groups in our study. Hospital stay did not differ between the two groups. Moreover, the overall length of stay was considerably longer than is typical for CABG, owing to the prolonged preoperative preparation period and the difficulty of providing patient education on the operation and wound care in our predominantly rural population. Consequently, the surgical team preferred to ensure patients’ full clinical stability before discharge.

Diakova et al. [10] reported that colchicine use was associated with lower levels of inflammatory markers, such as IL-10 and IL-6. In contrast, TNF-α and IL-1β levels did not differ significantly between the two groups. Their study also evaluated clinical complications, including pneumonia, sternal wound infections, and early postoperative bleeding [10]. Interestingly, pneumonia and wound infections were more common in the colchicine group, although the differences were not statistically significant [10]. However, the rate of early postoperative bleeding was significantly lower in the colchicine group [10]. In this respect, their findings appear to differ from ours. Although the differences were not statistically significant, wound complications and the need for surgical revision were less frequent in the colchicine group in our study. However, the range of inflammatory markers assessed in our study was limited, as inflammation was evaluated using only routinely measured parameters. To address this limitation, we assessed systemic inflammation using the systemic immune-inflammation index (SII), calculated from complete blood count parameters, which is a cost-effective and useful approach [24]. Although the differences were not statistically significant, NLR and PLR values were lower in the colchicine group.

Colchicine has other beneficial effects in patients undergoing CABG. Ponnana et al. [25] suggested that colchicine may reduce cardiovascular events and the incidence of atrial fibrillation (AF) after CABG. Diakova et al. [26] confirmed these findings regarding postoperative AF. Farzaneh et al. [27] also reported beneficial effects of colchicine in preventing AF after CABG. However, we were unable to assess postoperative AF because of limited access to the relevant medical records. Despite its limitations, our study reports findings that have not been described previously. Colchicine was found to be in relation with lower fresh frozen plasma transfusion requirements and shorter ICU stays, both of which are important clinical outcomes in patients undergoing CABG.

## 6. Limitations

The retrospective design and non-contemporaneous groups (2021–2022 vs. 2024–2025) may introduce calendar-time confounding. Importantly, there was no major change in transfusion, ICU, surgical, or perfusion protocols between the two periods. Nevertheless, differences in clinical decision-making and awareness of perioperative management cannot be excluded, particularly for the marked difference in FFP use without a difference in erythrocyte transfusion, which should be interpreted cautiously. ICU stay was shorter with colchicine, whereas total hospital stay was numerically longer; the latter includes a prolonged preoperative period and is longer than usually reported for elective CABG. Inflammatory markers were deliberately measured on postoperative day 5, because earlier values are strongly influenced by surgical variables such as cross-clamp and pump time. However, this choice meant that the opportunity to capture the early inflammatory peak was missed. Postoperative atrial fibrillation, pericarditis, and post-pericardiotomy syndrome could not be assessed because of limited access to data. The significant results are exploratory. A further limitation of this study is the lack of baseline data on heart failure, LVEF, arrhythmias, diabetes, NYHA class, and EuroSCORE II, which precluded a full assessment of group comparability for these parameters.

Strengths include the same surgical, anesthesia, perfusion, and ICU teams with standardized techniques, objective data from medical records, and relatively homogeneous elective on-pump CABG groups. The study also addresses clinical outcomes rather than biomarkers alone. Findings should be regarded as hypothesis-generating associations.

## 7. Conclusions

Perioperative low-dose colchicine was found to be in relation with a shorter ICU stay, lower fresh frozen plasma transfusion requirements, and reduced postoperative WBC counts in patients undergoing elective on-pump CABG according to our retrospective study. These findings suggest that colchicine may attenuate the postoperative inflammatory response and contribute to improved early clinical outcomes following CABG. However, colchicine use was not associated with significant differences in total hospital stay, erythrocyte transfusion requirements, CRP, NLR, PLR, albumin levels, wound complication rates, or the need for surgical revision. Although wound complications were numerically less frequent in the colchicine group, this difference did not reach statistical significance.

The observed reductions in ICU stay and fresh frozen plasma requirements represent clinically relevant findings that have not been extensively addressed in previous studies of colchicine in patients undergoing CABG. Nevertheless, given the retrospective design, relatively small sample size, non-contemporaneous study groups, and limited availability of detailed inflammatory and postoperative clinical data, these findings should be regarded as exploratory rather than as evidence of a causal relationship. Further large-scale, prospective, randomized controlled trials are required to confirm these results, clarify the underlying mechanisms, and determine whether perioperative colchicine should be incorporated into routine treatment protocols for patients undergoing CABG.

## Figures and Tables

**Table 1:** Continuous clinical outcomes and postoperative laboratory findings.

Variable	Colchicine	Control	Mean Difference (95% CI)	Test Statistic	*p* Value
Age, years	62.73 ± 8.94 (n = 81)	63.60 ± 10.34 (n = 70)	−0.88 (−3.99 to 2.24)	t = −0.56; df = 148	0.579
Intensive care unit stay, days	4.28 ± 1.95 (n = 81)	5.83 ± 3.04 (n = 70)	−1.54 (−2.38 to −0.71)	t = −3.65; df = 114.34	<0.001
Total hospital stay, days	27.10 ± 8.66 (n = 81)	24.53 ± 8.13 (n = 70)	2.57 (−0.14 to 5.28)	t = 1.87; df = 149	0.063
Erythrocyte transfusion, units	5.49 ± 2.70 (n = 81)	5.89 ± 2.85 (n = 70)	−0.39 (−1.28 to 0.50)	t = −0.87; df = 149	0.387
Fresh frozen plasma transfusion, units	3.49 ± 1.24 (n = 81)	8.13 ± 3.97 (n = 70)	−4.63 (−5.62 to −3.65)	t = −9.39; df = 80.58	<0.001
C-reactive protein, mg/L	101.84 ± 47.29 (n = 81)	101.81 ± 52.31 (n = 70)	0.03 (−15.99 to 16.05)	t = 0.00; df = 149	0.997
White blood cell count, ×10^9^/L	9.44 ± 3.31 (n = 81)	10.78 ± 4.04 (n = 70)	−1.34 (−2.52 to −0.16)	t = −2.24; df = 149	0.027
Neutrophil-to-lymphocyte ratio	5.89 ± 4.65 (n = 81)	5.29 ± 3.63 (n = 70)	0.59 (−0.76 to 1.95)	t = 0.87; df = 149	0.388
Platelet-to-lymphocyte ratio	164.83 ± 96.40 (n = 81)	179.77 ± 125.94 (n = 70)	−14.95 (−50.76 to 20.87)	t = −0.82; df = 149	0.411
Albumin, g/L	31.52 ± 3.45 (n = 81)	31.93 ± 3.67 (n = 70)	−0.41 (−1.56 to 0.74)	t = −0.71; df = 149	0.481

Values are mean ± SD. Mean differences are calculated as colchicine minus control. Welch’s *t* test was used for intensive care unit stay and fresh frozen plasma transfusion because Levene’s test indicated unequal variances; equal-variance Student’s *t* tests were used for the remaining variables. CI, confidence interval; CRP, C-reactive protein; NLR, neutrophil-to-lymphocyte ratio; PLR, platelet-to-lymphocyte ratio; SD, standard deviation.

**Table 2:** Categorical characteristics and postoperative complications.

Variable	Colchicine, n/N (%)	Control, n/N (%)	Statistical Test	*p* Value
Male sex	69/81 (85.0)	52/70 (74.3)	Pearson χ^2^ = 2.68	0.102
Wound complication	19/81 (23.5)	26/70 (37.1)	Pearson χ^2^ = 3.36	0.067
Surgical revision	5/81 (6.2)	4/70 (5.7)	Fisher’s exact test	1.000

Pearson’s chi-square test was used for sex and wound complication. Fisher’s exact test was used for surgical revision because 50% of expected cell counts were <5.

**Table 3:** Exploratory comparisons by sex within each treatment group.

Variable	Colchicine Group			Control Group		
	Male (n = 69)	Female (n = 12)	*p* Value	Male (n = 52)	Female (n = 18)	*p* Value
Age, years	62.06 ± 8.91	66.50 ± 8.50	0.113	63.67 ± 10.29	63.39 ± 10.79	0.921
Intensive care unit stay, days	4.33 ± 2.01	4.00 ± 1.60	0.588	5.79 ± 3.15	5.94 ± 2.80	0.853
Total hospital stay, days	27.28 ± 8.42	26.08 ± 10.28	0.663	25.15 ± 8.28	22.72 ± 7.59	0.277
Erythrocyte transfusion, units	5.67 ± 2.70	4.50 ± 2.58	0.168	5.67 ± 3.03	6.50 ± 2.20	0.291
Fresh frozen plasma transfusion, units	3.57 ± 1.24	3.08 ± 1.16	0.215	8.40 ± 4.45	7.33 ± 1.91	0.327
C-reactive protein, mg/L	104.93 ± 46.88	84.03 ± 47.70	0.159	107.67 ± 56.57	84.87 ± 33.08	0.111
White blood cell count, ×10^9^/L	9.59 ± 3.35	8.59 ± 3.03	0.340	10.85 ± 4.23	10.57 ± 3.51	0.801
Neutrophil-to-lymphocyte ratio	6.05 ± 4.81	4.95 ± 3.61	0.456	5.16 ± 3.57	5.67 ± 3.88	0.613
Platelet-to-lymphocyte ratio	165.47 ± 97.56	161.16 ± 93.42	0.887	182.53 ± 128.54	171.82 ± 121.31	0.758
Albumin, g/L	31.43 ± 3.56	32.00 ± 2.83	0.604	31.77 ± 3.88	32.39 ± 3.03	0.541

Values are mean ± SD. *p* values were obtained using independent-samples *t* tests. Welch’s correction was applied only when Levene’s test was significant.

**Table 4:** Pearson correlation matrix for the colchicine group.

Variable	1	2	3	4	5	6	7	8	9	10
**1. Age**	1.000	—	—	—	—	—	—	—	—	—
**2. ICU stay**	−0.043 (0.705)	1.000	—	—	—	—	—	—	—	—
**3. Hospital stay**	0.179 (0.112)	0.472 (<0.001)	1.000	—	—	—	—	—	—	—
**4. Erythrocyte**	0.067 (0.557)	0.500 (<0.001)	0.470 (<0.001)	1.000	—	—	—	—	—	—
**5. FFP**	−0.113 (0.318)	0.335 (0.002)	0.304 (0.006)	0.529 (<0.001)	1.000	—	—	—	—	—
**6. CRP**	0.201 (0.074)	0.112 (0.319)	0.399 (<0.001)	0.204 (0.068)	0.125 (0.268)	1.000	—	—	—	—
**7. WBC**	−0.146 (0.196)	0.137 (0.223)	−0.160 (0.153)	0.062 (0.580)	0.003 (0.976)	−0.009 (0.935)	1.000	—	—	—
**8. NLR**	0.203 (0.071)	0.196 (0.080)	0.088 (0.437)	0.239 (0.032)	−0.007 (0.950)	−0.011 (0.926)	0.422 (<0.001)	1.000	—	—
**9. PLR**	0.286 (0.010)	0.151 (0.180)	0.084 (0.454)	0.014 (0.898)	−0.099 (0.378)	0.033 (0.769)	0.446 (<0.001)	0.777 (<0.001)	1.000	—
**10. Albumin**	−0.201 (0.073)	−0.282 (0.011)	−0.389 (<0.001)	−0.455 (<0.001)	−0.175 (0.118)	−0.319 (0.004)	0.009 (0.936)	−0.217 (0.052)	−0.117 (0.299)	1.000

Cells show Pearson’s r with the two-sided *p* value in parentheses; only the lower triangle is displayed. Pairwise deletion was used. Abbreviations: CRP, C-reactive protein; FFP, fresh frozen plasma; ICU, intensive care unit; NLR, neutrophil-to-lymphocyte ratio; PLR, platelet-to-lymphocyte ratio; WBC, white blood cell count.

**Table 5:** Pearson correlation matrix for the control group.

Variable	1	2	3	4	5	6	7	8	9	10
**1. Age**	1.000	—	—	—	—	—	—	—	—	—
**2. ICU stay**	0.091 (0.454)	1.000	—	—	—	—	—	—	—	—
**3. Hospital stay**	0.128 (0.292)	0.550 (<0.001)	1.000	—	—	—	—	—	—	—
**4. Erythrocyte**	0.109 (0.370)	0.252 (0.035)	0.227 (0.059)	1.000	—	—	—	—	—	—
**5. FFP**	0.040 (0.744)	0.260 (0.030)	0.193 (0.110)	0.486 (<0.001)	1.000	—	—	—	—	—
**6. CRP**	0.209 (0.082)	0.167 (0.168)	0.012 (0.918)	−0.009 (0.940)	−0.050 (0.681)	1.000	—	—	—	—
**7. WBC**	0.030 (0.804)	0.429 (<0.001)	0.274 (0.022)	−0.058 (0.633)	0.149 (0.217)	0.098 (0.418)	1.000	—	—	—
**8. NLR**	0.297 (0.013)	0.401 (<0.001)	0.333 (0.005)	0.261 (0.029)	0.336 (0.004)	0.271 (0.023)	0.506 (<0.001)	1.000	—	—
**9. PLR**	0.279 (0.019)	0.156 (0.196)	0.207 (0.086)	−0.032 (0.790)	0.332 (0.005)	0.114 (0.348)	0.415 (<0.001)	0.531 (<0.001)	1.000	—
**10. Albumin**	−0.226 (0.060)	−0.253 (0.035)	−0.131 (0.280)	−0.419 (<0.001)	−0.457 (<0.001)	−0.225 (0.062)	−0.082 (0.499)	−0.298 (0.012)	−0.278 (0.020)	1.000

Cells show Pearson’s r with the two-sided *p* value in parentheses; only the lower triangle is displayed. Pairwise deletion was used. Abbreviations: CRP, C-reactive protein; FFP, fresh frozen plasma; ICU, intensive care unit; NLR, neutrophil-to-lymphocyte ratio; PLR, platelet-to-lymphocyte ratio; WBC, white blood cell count.

## Data Availability

The original contributions presented in this study are included in the article. Further inquiries can be directed to the corresponding author.
